# Social integration as a determinant of inequalities in green space usage: Insights from a theoretical agent-based model

**DOI:** 10.1016/j.healthplace.2021.102729

**Published:** 2022-01

**Authors:** Stefano Picascia, Richard Mitchell

**Affiliations:** MRC/CSO, University of Glasgow, Social and Public Health Science Unit, Berkeley Square, 99 Berkeley Street, Glasgow, G3 7HR, Scotland, United Kingdom

**Keywords:** Simulation, Urban green spaces, Agent based model, Class segregation, Scotland, Microsimulation

## Abstract

Visiting urban green spaces (UGS) benefits physical and mental health. However, socio-economic and geographical inequalities in visits persist and their causes are under-explored. Perceptions of, and attitudes to, other UGS users have been theorised as a determinant of visiting. In the absence of data on these factors, we created a spatial agent-based model (ABM) of four cities in Scotland to investigate intra- and inter-city inequalities in UGS visiting. The ABM focused on the plausibility of a ‘social integration hypothesis' whereby the primary factor in decisions to visit UGS is an assessment of who else is likely to be using the space. The model identified the conditions under which this mechanism was sufficient to reproduce the observed inequalities. The addition of environmental factors, such as neighbourhood walkability and green space quality, increased the ability of the model to reproduce observed phenomena. The model identified the potential for unanticipated adverse effects on both overall visit numbers and inequalities of interventions targeting those in lower socio-economic groups.

## Highlights

• Differentials in urban green space visitation can emerge from inter-group (in)tolerance.
• Socio-spatial segregation is a possible “hidden” determinant of inequality in UGS usage.
• Interventions based on simply promoting green space use can have unintended consequences.

## 1 Introduction

This paper describes an agent-based model (ABM) built to test theory about the determinants of contact with nature and drivers of socio-economic and geographical inequalities in visiting urban green space (UGS). First, we set out the problem and specify our questions. We then describe the modelling approach and the model itself, before showing how the model answers our questions and considering the implications.

Contact with nature is positively associated with a variety of indicators of physical (White et al., 2019) and mental (Ward Thompson et al., 2016; Roe et al., 2013) well being, and may even act to constrain socio-economic health inequalities (Mitchell and Popham, 2008). However, engagement with UGS is, itself, strongly socially patterned. Evidence suggests less advantaged populations are, in general, markedly less likely to visit natural environments, including UGS (Dallimer et al., 2014; Bell et al., 2014; Boyd et al., 2018; Pitt, 2019).

**Fig. 1:**
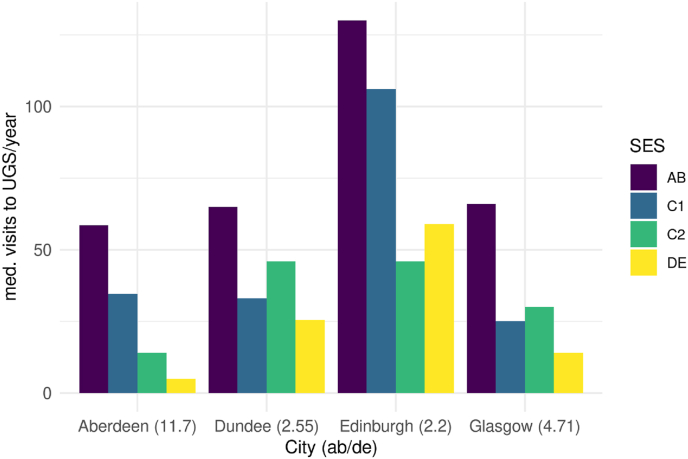
Median visits to UGS per year by SES, by city (SPANS, 2014; *estimated*). The number in parenthesis is a measure of inequality between the two extreme groups.

Fig. 1 plots, for four key Scottish cities, frequency of visits to UGS against socio-economic status (SES) identified by a four-category occupational grade from AB (the highest), to C1, C2 and DE (the lowest). Data stem from Scotland's People and Nature Survey (SPANS), a repeat cross-sectional, representative measure of Scots' engagement with nature and the outdoors. (SPANS, 2014). As expected, an intra-city socio-economic inequality in visits is evident in all the cities. However, Fig. 1 also reveals inter-city differences. First, people in Edinburgh (apart from those in the ‘C2′ category) visit UGS at least twice as much as those in the corresponding SES group in any other city. Second, Edinburgh displays the lowest socio-economic inequality in the frequency of UGS visits: those in the ‘AB’ group visit parks a little more than twice as often than those in ‘DE’, compared with almost 12 times in Aberdeen and almost 5 in Glasgow. These intra- and inter-city differences in UGS visiting persist on adjustment for demographic factors in a regression model (Section 1, supplementary material). This paper focuses on these intra- and inter-city differences as a means to deepen our understanding of influences on, and inequalities in, visits to UGS.

## 2 The socio-spatial determinants of visiting UGS

While the literature offers abundant evidence for the benefits of contact with nature, not much has been uncovered about its determinants (Dallimer et al., 2014; Hitchings, 2013). Quantitative evidence suggest that the most obvious environmental factors, such as quality and proximity of UGS, are comparatively weak influences (Schipperijn et al., 2010). More nuanced and difficult to capture factors may be more important: childhood and early life experience of natural space may play an important role (Thompson et al., 2008), low orientation to nature, lack of interest and lack of time are more likely to be reported as reasons for not visiting (Boyd et al., 2018).

Qualitative literature, on the other hand, has investigated both attitudinal and environmental influences, and has begun to explore how these might interact. Jones et al. (2008) and Seaman et al. (2010) found that individuals’ decisions about whether to visit a particular park were strongly influenced by *who else is, or might be, there*. Perceived differences with other users, particularly in terms of SES, affected the reported willingness of respondents to visit a certain green space, with some of the interviewees identifying the presence of *neds* (a derogatory Scottish term referring to someone of a low social standing) as cause for avoiding specific spaces. Similarly, Gibson (2018) found the presence of people of “similar social class” as a factor considered by older Australian adults when deciding whether to visit UGS.

This influence is intriguing, because implicit preferences for the company of certain people more than other could be powerful “hidden” driving factors of the variation observed in visits to UGS, as they imply that individuals’ visiting behaviour and experience will shift and adjust according to that of their fellow urban residents. The morphology of the city and the distribution of social groups would also play a role, as who we encounter when we visit an UGS depends ultimately on where the UGS is located and the kinds of people who live nearby or are willing and capable of getting there. The way the presence of others affects our experience, on the other hand, is influenced by a large and nuanced set of psychological, social, and environmental factors, not least the state of social integration in society at large (Seaman et al., 2010).

Relationships between individual visiting choices, urban morphology, and which kinds of people visit (which) parks are complex and evolving – a system shaped by a plurality of factors and circumstances in combination and interactions. These are very difficult to quantify in a static regression model (Galea et al., 2010). To explore these ideas, given that several variables and relationships within the system are theorised rather than measured, we need an approach that is also able to blend more and less reliably quantified information, with theory. In situations like this ABMs can be valuable tools (Andersson et al., 2014; Marshall, 2017).

## 3 Agent-based models and research questions

An ABM is a computer simulation of the behaviours and interactions of autonomous *agents* (in this case representing individuals) set within their environment (in this case the four main Scottish cities). As the individuals act, they also interact and may modify their own behaviours and those of others in response. ABMs enable us to test the explanatory capabilities of our theories where hard, robust data may be patchy, difficult to obtain or entirely unavailable (Silverman et al., 2021).

In this study, we created a stylised agent-based model^1^ designed to offer a candidate explanation of the UGS visting patterns observed in Scotland. Specifically we wished to explore to what extent – given each Scottish city's social composition, residential distribution and placement of UGS – the inter-group preferences hinted at by Seaman et al. (2010) could be driving forces behind the observed intra and inter urban difference in green space use. We encoded the basic assumption of the “social integration” *quasi-theory* — that people decide whether to visit an UGS based on their preference on the social composition of others in the park — and determined whether the resulting model was able to reproduce the salient patterns observed in Fig. 1: intra-city socioeconimic inequality, inter-city differences with higher and less unequal visitation in Edinburgh. If it could, the inter-group preference mechanism implemented in the model could be considered plausible parts of the causal chain that produces the phenomenon. (Epstein, 1999, 2006; Edmonds et al., 2019).

Our research questions were:

1. Can individuals' preferences for what kinds of other people share their UGS, on their own, account for the intra- and inter-city differences in UGS visiting?
2. What role do other morphological characteristics such as walkability and park quality play in establishing intra- and inter-city differences in UGS visiting?
3. How sensitive to parameter choice is the emergence of the intra- and inter-city differences in UGS visting?
4. What are the implications for those promoting and researching UGS use?

## 4 Modelling approach

Below we set out the key variables, parameters and thinking in the ABM. A full ODD description of the model is provided in the supplementary materials.

### 4.1 Primary mechanism

Building on the findings described in the introduction, the model's main assumption was that the decision whether to visit a UGS is influenced primarily by the agent's assessment of what kinds of people were in the space when they visited a previous time, and that this assessment was focused on SES. To operationalise this, we drew on sociological theory which suggests that, in hierarchical societies, those belonging to higher prestige groups prefer to mix with others who are similar, but to distance (ideally, culturally and physically) from the lower groups (Bourdieu, 1979). This is referred to as a *homophilic preference*, a desire to be with similar types of people. Geography and Planning literature offers abundant evidence of this being acted out in physical space (Massey and Denton, 1988; Reardon and Bischoff, 2011; Atkinson, 2016; Osborne, 2014).

Rather less research is available about the preferences of those in lower SES groups and the findings are mixed. Some research finds those of lower SES are also better inclined towards people in the same SES groups (Côté et al., 2017). However, recent research using novel data sources, such as credit card transaction data, suggests that for shopping and recreation, people of lower SES like to spend a substantial amount of time in areas populated by those from higher SES groups (Dong et al., 2020). This is referred to as a heterophilic preference.

Given the conflicting evidence, we modelled the top two socioeconomic groups (AB, C1) as homophilic, and assume at least a portion of the bottom two (C2, DE) as being heterophilic, preferring the company of agents of higher socio-economic status. These preferences are implemented in the form of tolerance thresholds (described in detail in 4.4) following an approach popularised by Thomas Schelling with his study of the socio-spatial problem *par excellence*, ethnic segregation (Schelling, 1971a), and adopted in several models of inter-group relations (Portugali et al., 1997; Jager and Amblard, 2005; Alizadeh et al., 2014).

### 4.2 Agent characteristics and initial probability to visit UGS

All agents are endowed with age, place of residence, socio-economic status, derived from the 2011 UK Census, plus a tolerance threshold towards members of the ‘other’ socio-economic group (i.e. the one that they do not belong to) (Table 2).

Upon initialization the initial probability of each agent to visit a green space (*p*_*i*_) is set, drawn from a Normal distribution with *μ* = 0.07 (equivalent to a visit every ~ 14 days, the overall average in SPANS) and *σ* = 0.025. We assume that the initial probability decreases with age, again consistently with our finding from the SPANS survey. We assume that all UGS visitation takes place on foot, the initial probability is therefore halved for those living far away from any UGS, in line with findings from several studies suggesting that distance is relevant in people's decision to visit a green space only when it exceeds a walk of around 20/25 min. Surveys show that those who own dogs visit green spaces much more frequently (Burnett et al., 2021), we therefore assume that a dog owner visits a green space with a minimum daily probability of *p* = 0.33, assuming the responsibility for dog walking being shared in the household. The proportion of agents who own dogs is set at 24% in all cities, with SES differences derived from real data (Marsa-Sambola et al., 2016). Finally, a form of “localised social influence” was assumed. Agents tended to conform to an implicit norm resulting from the observation of the behaviour of neighbouring agents of similar age and social status: when large differences in the practice of visiting green spaces existed with this ‘reference group’, the agent were assumed to slightly modify their behaviour towards that of the group (exact process is detailed in the ODD document, Supplementary material).

### 4.3 Environment

**Fig. 2:**
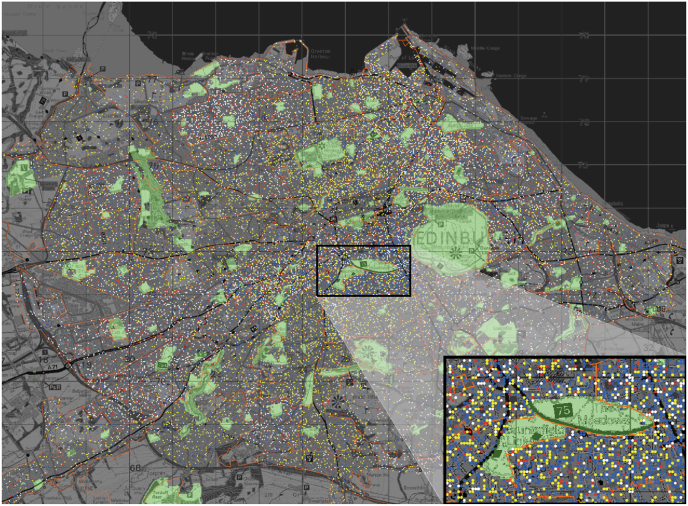
The City of Edinburgh as represented in the model. The dots represent individuals, colour coded by their SES. yellow = AB, red = C1, blue = C2, white = DE. The green spaces are explicitly represented; the orange boundaries designate ‘neighbourhoods', based on the Postcode sector Census subdivision.

The agents existed in spatially explicit representations of Edinburgh (Fig. 2), Glasgow, Aberdeen, and Dundee. The models included the cities' geography, their public green spaces, and their 16–75 population at a 1/10 scale. Urban morphology is also known to influence residents' interaction with different amenities in a city. Empirical evidence for the impact of built environment on behaviours such as physical activity is abundant (Herrick, 2009). The line of thought originating with the morphological determinism of Jane Jacobs (1961) argues that dense, small-scale, mixed use urban areas afford walking and the development of a street life. Jacobs specifically examined parks as both barriers and connectors between communities. Certain aspects of the urban morphology she wrote about now find their expression in measures of the ‘walkability’ of streets and neighbourhoods and, whilst the specific relationship between walkability and engagement with UGS has been examined to a lesser extent, a positive impact is documented in recent literature. For example, a study found that walkability elements influenced the probability of greenspace visitation in Tucson, Arizona (Zuniga-Teran et al., 2019) and a London-based study (Clary et al., 2020) linked walkability with increases in both physical activity and green space usage. We know that the walkability of streets is also related to socio-economic position of the neighbourhood (Macdonald et al., 2016).

We included the influence of the morphological element of walkability as an “effect modifier” of the agents' behaviour. We assigned a walkability attribute to each area of the four cities, based on an index composed of dwelling density and street intersections, expressed in quartiles and calculated by Lower Layer Super-Output Area level (Macdonald et al., 2016). We express walkability as a constant, *w*, which affects an agent's probability of visiting a green space. We set *w* = 0.5 for areas in Walkability Quartile 1, *w* = 0.66, *w* = 1 and *w* = 2 for areas in Q2, Q3 and Q4 respectively. Table 1 summarises all location attributes. Another constant impacting all agents in the model was the weather. There are differences in the amount of sunshine across Scottish cities, and evidence for an impact of the weather on visiting behaviour is consistent and strong. We obtained the average number of sunny hours per year from https://www.currentresults.com/Weather/United-Kingdom/annual-sunshine.php#d. Glasgow has the lowest amount of sunshine (1265 h per year), Dundee the highest at 1564 h. Assuming poor weather discourages everyone from going out, we set *s* = 1 for agents in Dundee, *s* = 0.80 in Glasgow, *s* = 0.88 in Edinburgh and Aberdeen.

**Table 1:** Location attributes.

variable	description	type	range	source	location type
*z*	size in ha	int	0-500	OS Green space	park
*q*	quality	cat	low, medium, high	hypothesis	park
*n*	neighbourhood	string		Census 2011	residential
*w*	walkability	real	0.5; 0.66; 1; 2	(Macdonald et al., 2016)	residential

We considered UGS size and quality. Whilst evidence for the influence of both on visiting behaviour is inconsistent, there is some evidence to suggest that larger UGS are more attractive (Sugiyama et al., 2010), therefore, each UGS had a ‘catchment’ proportional to its size. The implication of this is that agents may have more than one park within their reach and, if they choose to visit an UGS, they must decide which one. Finally, there is evidence that green spaces in the deprived areas of Glasgow are more likely to be poorly maintained than those in affluent areas (Ellaway et al., 2007). We generalised this finding and assumed that smaller UGS (size below the city average) in deprived areas were more likely to be of poorer quality. To avoid introducing a straightforward penalisation to residents of deprived areas, we also assume that people of higher SES are more likely to avoid green spaces in below than average maintenance state than those of lowers SES. Details of the implementation are offered in the ODD document, Supplementary Material.

Upon initialization, the urban environment was generated from Ordinance Survey datasets; agents were generated from UK Census datasets and assigned to a random location within their postcode sector of residence. Postcode sectors house around 5000 residents, represented in the model as 500 agents. The modelled population was of 11 757 agents in Aberdeen, 10 791 in Dundee, 34 280 in Edinburgh and 44 919 in Glasgow.

### 4.4 Simulation flow

The model simulated 1460 days, or 4 years of visiting. The main output of the ABM was the number of visits on the part of each modelled agent over this period.

**Fig. 3:**
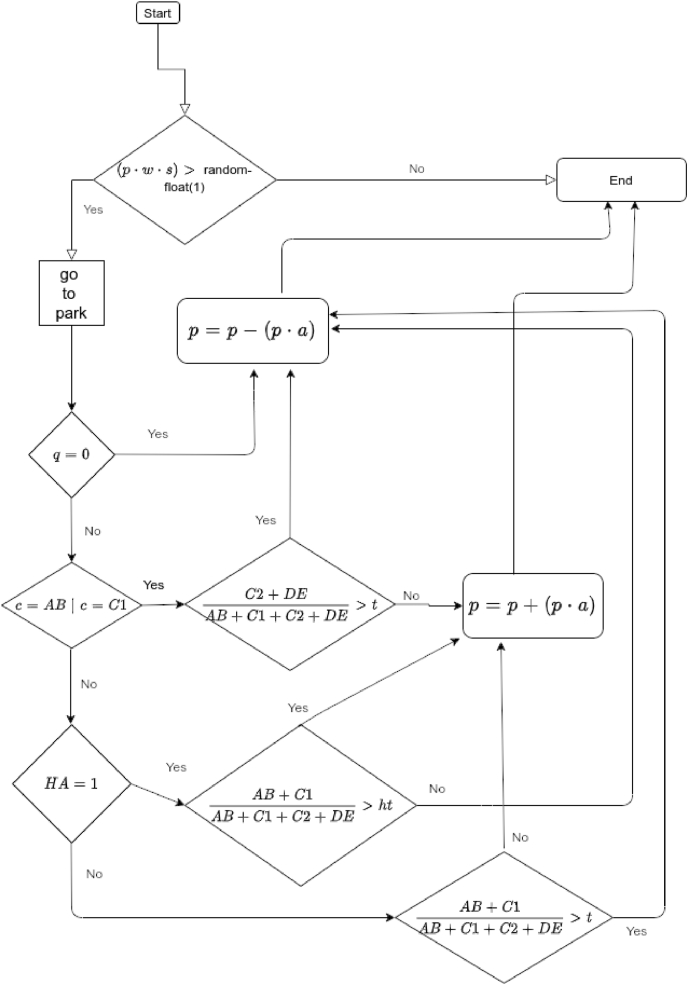
Simulation step.

During each simulated day agent *i* visits the first green space in their list of accessible spaces *g* with probability *p*_*i*_ × *w* × *s*. Those who visit an UGS evaluate a subset of other visitors based on their homophily /heterophily preferences, implemented as “dissonance thresholds”. We denoted an individual level attribute, *t*, as the maximum proportion of individuals of the *other group* that one is willing to accept. We assumed that agents of a higher status (AB and C1) prefer to share the UGS with agents similar to themselves, therefore agents from the lower SES (C2 and DE) constitute the ‘other’ group. If the proportion of agents of the other group within the UGS exceeded *t*, the probability of visiting any green space the subsequent day (*d*) was reduced of factor *a*, so that *p*_*i*,*d*+1_ = *p*_*i*,*d*_ − (*a* × *p*_*i*,*d*_). If the agent has more than one UGS within their reach, they also move the unsatisfying UGS to the bottom of the list *g*, so that the next time they will try a different space. In contrast, if the number of ‘others' in the UGS does not exceed the threshold, the agent increase their probability of visiting again, and to the same space: *p*_*i*,*d*+1_ = *p*_*i*,*d*_ + (*a* × *p*_*i*,*d*_). Agents in groups C2 and DE behave the same way: they increase the likelihood of visiting an UGS again if the proportion of agents of the other group doesn't exceed *t*. However, a proportion *h* of agents in groups C2 and DE was assumed to be *heterophilic*: they prefer the company of agents of the upper SESs rather than of their own. For these agents, those in their own SES constitute the “other group”. We therefore defined an individual attribute, *ht*, as the minimum proportion of agents of SES AB or C1 that an heterophilic agent of SES C2 or DE will accept.^2^ Table 2 summarises all individual attributes, Table 3 lists model-wide parameters, Fig. 3 illustrates the process.

**Table 2:** Agent attributes.

variable	description	type	range	source	held by
*c*	socio economic status	string	AB, C1, C2, DE	2011 Census	all agents
*y*	age	int	16–75	2011 Census	all agents
*g*	UGS accessible	list	{ … }	GIS	all agents
*p*	prob. visiting UGS	real	0–1	hypothesis	all agents
*t*	homophily threshold	real	0–1	hypothesis	agents with *HA* = 0
*ht*	heterophily threshold	real	0–1	hypothesis	agents with *HA* = 1
*HA*	heterophilic agent	bool	0,1	hypothesis	agents with *c=C2|DE*
*v*	# visits to UGSs	int	0–1460^a^	model output	all agents
*dg*	dog owner?	bool	0,1	(Marsa-Sambola et al., 2016)	all agents

a 4 years = 1460 days, theoretical maximum number of visits.

**Table 3:** Model environment variables.

variable	description	type	range	source
*s*	weather	real	0–1	Current results website a
*h*	proportion of agents of C2 and DE SES with *HA* = 1	real	0.33; 0.5; 0.66; 1	hypothesis
*a*	probability adjustment factor	real	0.25	hypothesis^b^

a https://www.currentresults.com/Weather/United-Kingdom/annual-sunshine.php.

b An analysis of model sensitivity to parameter *a* is offered in the Supplementary material.

## 5 Results: emergence of intra- and inter-city differences

We assess the ability of the model outlined above to produce the constituent aspects of the phenomenon observed in the empirical data: a) socio-economic differentials, whereby agents in the more advantaged socio-economic groups visit green spaces more than those in the less advantaged ones (Equation (1) below); b) inter-urban differences, whereby agents in Edinburgh visit green spaces more than those in all the other cities (Equation (2)) and with the lowest inequality between agents of the AB and DE SES (Equation (3)). Formally, if all three conditions below are verified *∀city* ∈ {*glasgow*, *aberdeen*, *dundee*}

(1) $$med{\left(v_{AB}\right)}_{city}>med{\left(v_{DE}\right)}_{city}\;\;\;\;;\;\;\;\;med{\left(v_{AB}\right)}_{edinburgh}>med{\left(v_{DE}\right)}_{edinburgh}$$

(2) $$V_{edinburgh}>V_{city}\;where\;V=\sum_{i=1}^{N}v$$

(3) $$\frac{med{\left(v_{AB}\right)}_{edinburgh}}{med{\left(v_{DE}\right)}_{edinburgh}}<\frac{med{\left(v_{AB}\right)}_{city}}{med{\left(v_{DE}\right)}_{city}}$$

where *v*_*i*_ is the total number of UGS visits an agent accrued in the course of the simulation and *N* the total number of agents in a city.

The parameters driving the model are the homophily and heterophily thresholds, *t* (the tolerance of homophilic agents towards those of the ‘other’ group) and *ht* (the minimum proportion of agents of higher SES that satisfies heterophilic agents of the lower SES) respectively, and the proportion of agents of the lower SES who are heterophilic - seeking the company of agents of higher SES, *h*.

Whilst the theory is a textual description of how and why people might behave, an ABM needs numerical rules via which agents can make their assessments and behavioural choices. There is no data to guide this parameterisation and, indeed, understanding whether and how the parameters of *t*, *ht*, and *h* affect the model was a key aim of the study. We therefore undertook a robust process of parameter testing and comparison. Values of *t* and *ht* were drawn from Normal distributions with *μ* between 0.3 and 0.7, *σ* = 0.05, tested at 0.1 intervals. Four values of *h* were also tested, 0.33, 0.5, 0.66, 1 giving rise to 100 possible combinations for each city. All combinations were repeated 10 times and the average taken to discount the stochasticity embedded in the model (see ODD, Supp.mat). Factor *a* was set at 0.25, the model was tested for sensitivity to different values of *a*, the details are available in the Supplementary material section.

### 5.1 Testing the social integration hypothesis

Research question 1 (Can individual's preferences for what kinds of other people share their UGS, on their own, account for the intra- and inter-city differences in UGS visiting?) was explored by model runs in which all urban morphological factors were ignored (except distance to UGS), setting walkability *w* = 1 in all locations and ignoring the quality of parks.

**Fig. 4:**
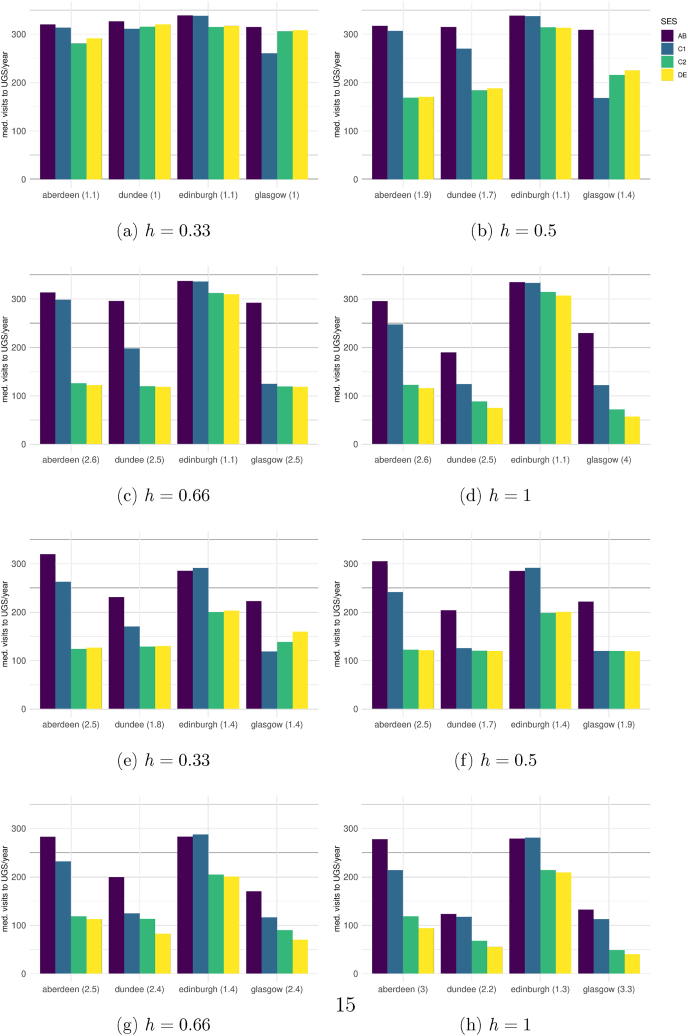
Median visits per SES per city at simulation end (*d* = 1460) for different values of *h* with *t* = *ht* = 0.5. Inequality shown in parentheses, expressed as the *AB*/*DE* proportion. Figures a–d: scenario with social component only. Figures e–h: scenario with social component and environmental constraints.

Diagrams a-d in Fig. 4 show the results of a batch of model runs in which, for simplicity, we assume *t* = *ht* = 0.5, meaning that all agents are satisfied if the majority of other UGS visitors belong to the social group they wish to see.

When only a minority of agents in the C2 and DE groups are heterophilic (*h* < 0.5) the final state of the simulation deviates from the observed situation: the model produces high rates of UGS visitation and low inequality in *all* cities (Fig. 4a). This is because Scotland's cities are socio-spatially segregated, but UGS is evenly distributed, a situation which makes it possible for agents of all socioeconomic groups to sort within UGSs. SES-segregated UGS then emerge, and all agents can meet a majority of similar agents, increasing their willingness to visit.

On the contrary, for values of *h* ≥ 0.5 (the assumption of half or more agents in the lower SES preferring green spaces populated by a majority of agents of higher status, figures b–d), intra-city differences emerged, similar to those observed in Fig. 1. In all cities agents in the upper SES groups visited consistently more than others. Inter-city differences also emerged, Edinburgh exhibited both the highest number of overall visits and the lowest inequality between agents in the top and bottom social groups. This set of experiments confirmed that, for a subset of the parameter space, the theorised mechanism is *sufficient* to generate an approximation of the real-world pattern.

An advantage of ABMs is their ability to give insights not only into what patterns emerge, but also *why*. The different outcomes within and between in the four cities stemmed from the differences in social composition and spatial distribution of the population within them. We observed that the factors driving emergence in this model run were, for the most part: (1) the number of agents in higher social grades, and (2) their level of spatial segregation. Broadly, when in a city there are many, well spatially distributed, agents of grade AB and C1, they are more likely to encounter similar agents in UGS, therefore they will be satisfied often with the social composition within the park. Hence their tolerance threshold *t* will rarely be exceeded and their probability of visiting again will increase. At the same time, the willingness to visit of the heterophilic portion of agents of the lower SES will *also* increase, as they encounter agents of higher groups often enough, thanks to the higher groups’ abundance and availability across the city. This is the situation in Edinburgh, a city with a high proportion of agents of the AB and C1 SES and a lower level of socio-spatial segregation (refer to the Supplementary Material for segregation levels and a breakdown of SES within cities). Glasgow, in contrast, has fewer agents in the top two socio-economic groups, and they are more spatially segregated. The segregation (i.e. that they tend to live in the same parts of the city) means agents from higher groups do often encounter similar agents frequently, but the heterophilic agents of the lower social groups seldom encounter enough agents from higher social groups in the green spaces they access. Their propensity to visit UGS therefore decreases. This produces a situation in which inequality in UGS visits is higher, and the overall number of visits stays relatively low.^3^

### 5.2 Impact of environmental factors: walkability, green space quality

To explore research question 2 (What role do other morphological characteristics such as walkability and park quality play in establishing intra- and inter-city differences in UGS visiting?) we added the environmental factors, walkability and UGS quality, to the baseline model (Diagrams e − h in Fig. 4). The intra- and inter-city patterns also emerged, but this time also when only one in three agents from the lower SES groups are heterophilic (values of *h* = 0.33, Fig. 4e).

The effect of walkability was to increase inequality in every city except Dundee. We explored the reasons for this. In the other cities, the lower SES groups tend to concentrate spatially in peripheral estates that have low levels of walkability. This diminishes their propensity to visit UGS. In Dundee however, it's higher SES groups who tend to live on the city fringes and experience lower walkability. Assuming that higher proportions of the lower SES group agents are heterophilic increased inequality in all cities apart from Edinburgh and this was particularly apparent when walkability and park quality were added (e-h, Fig. 4). Here, the twin assumptions of smaller parks in deprived areas being under-maintained and agents from higher SES groups being less likely to visit under-maintained spaces, forced more lower SES agents to travel further afield (seeking the higher SES agents they wanted to be with). This, in turn, increased their proportion in other green spaces which, in turn, led some agents from higher SES groups to withdraw, resulting in fewer UGS visits overall and higher inequality. This finding was a useful illustration of the “systemic” nature of UGS visiting and the ability of an ABM to capture it.

### 5.3 Effect of different thresholds

**Fig. 5:**
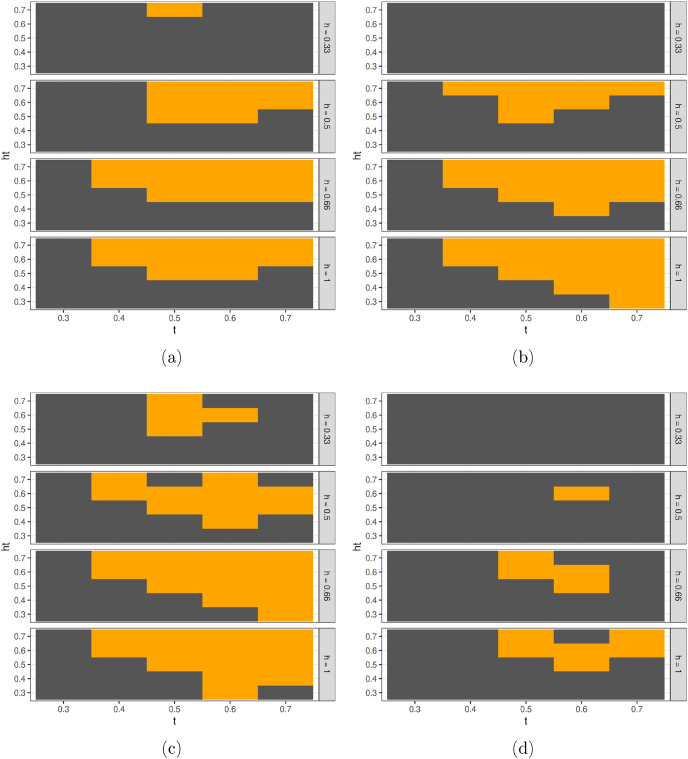
In orange, combinations of *t*, *ht* and *h* for which equations (1), (2), (3)) in Section 5 are verified (inter- and intra-city inequalities jointly emerge in the model). In (a) only the social component is implemented, in (b) walkability is added, in (c) we include green space quality. Plot (d) shows a quasi-null model in which, within each city, the residence of agents is randomised, removing the effect of socio-spatial segregation.

To explore research question 3 (How sensitive to parameter choice is the emergence of the intra- and inter-city differences in UGS visiting?) we dropped the assumption that all agents were satisfied if the majority of other park-goers belonged to the social group they wish to see (i.e. *t* = *ht* = 0.5) and instead tested all combinations of tolerance thresholds. In doing so, we derived the full set of parameter combinations for which the conditions in Eqs. (1), (2), (3)) are verified. Doing this reveals how strict our assumptions about behaviour and thresholds need to be for the intra- and inter-city differences in UGS to emerge from the model. Fig. 5 shows the results. Shaded in orange are all combinations of *t* (homophily), *ht* (heterophily) and *h* (proportion of agents of lower SES that seek the company of agents of higher SES) for which the phenomena of socio-economic gradients and the Edinburgh effect jointly emerged. In general, assuming more tolerance of higher SES agents towards others, less tolerance of those in lower SES groups who are heterophilic towards those with their own status, and higher fractions of heterophilic agents overall, improved the chances the model would generate the intra- and inter-city differences. The inclusion of morphological factors progressively increased the portion of the parameter space which produces the observed pattern. The greater amount of orange shading on Fig. 5b (walkability added) and 5c (green space quality added) reveals that less strong behavioural assumptions are needed to produce the observed dynamics when the environment — walkability and UGS maintenance differences — were taken into consideration. Fig. 5d shows the results of model runs in which the effect of socio-spatial segregation (i.e. residential clustering by SES group) was removed by randomising the agents’ places of residence within each city. In these runs the intra- and inter-city differences in UGS visiting emerged much less frequently, and only for very specific combinations of preferences. This suggests that, within the perimeter of our assumptions, urban segregation is also a substantial factor in the emergence of inequalities in UGS visiting.

**Fig. 6:**
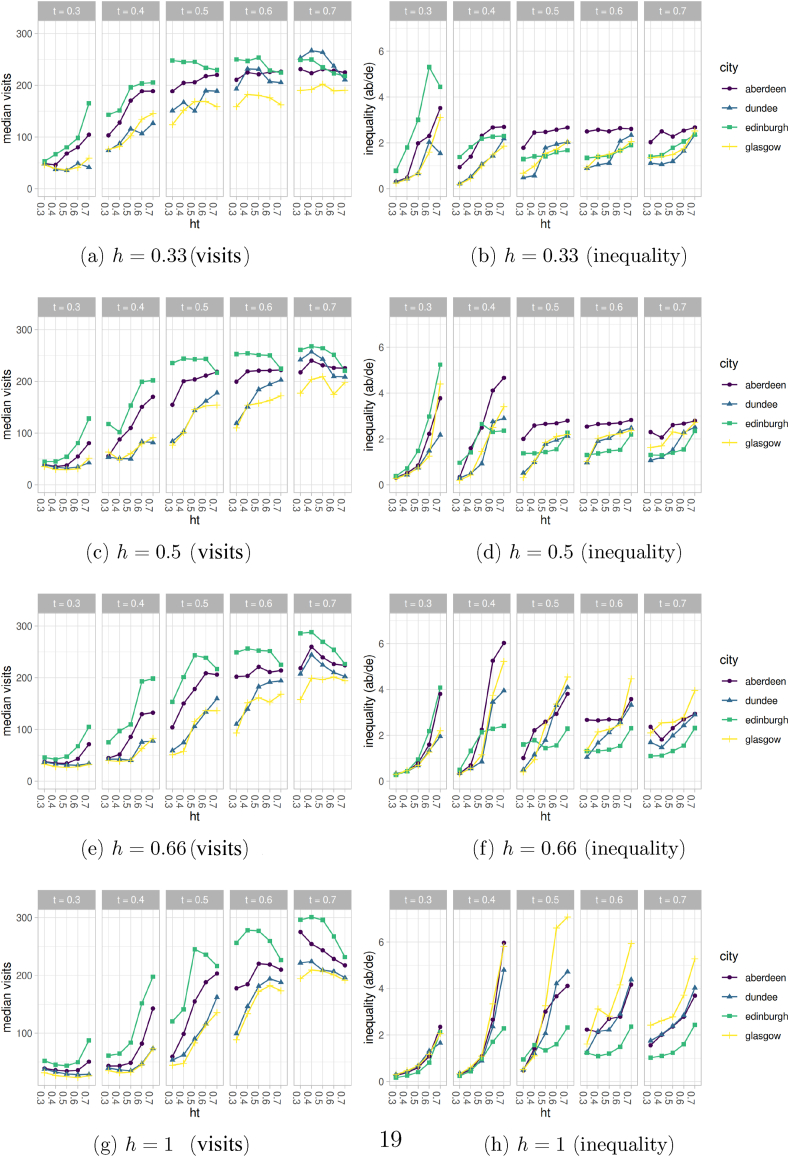
Median visits and inequality (AB/DE) by city at simulation end (*d* = 1460) for all tested combinations of *t*, *ht* and *h*.

Finally, we examined the interaction between the three key variables *t* (the tolerance of homophilic agents towards those of their *other* group), *ht* (the proportion of agents of higher SES that heterophilic agents of lower SES want to see in an UGS) and *h* (the proportion of lower SES agents who are heterophilic) to clarify the dynamics of the model. The plots in the left-hand column of Fig. 6 show the median number of visits to UGS that emerged from the model, whilst those in the right-hand column show the inequality between the top and bottom socio-economic groups, under different combinations of *t* and *ht*. The rows in the panel of graphs represent different values of *h*, the proportion of lower SES agents who are heterophilic.

Fig. 6 shows that an increase in *t* always resulted in an increase in visits to UGS, in all cities. This is expected: if homophilic agents accept the presence of a higher number of agents belonging to the *other* group, they will visit green spaces more. Consequently, lower SES agents who are heterophilic will encounter more higher SES agents within UGS, resulting in their *ht* threshold being met more frequently, and so they too visit more often. Fig. 6 also shows that increasing the level of *ht* almost always led to an increase in the overall number of visits to UGSs (left-hand plots). At first sight, this seemed counter-intuitive. Increases in *ht* might imply that fewer agents from low SES groups will visit UGSs because they want spaces with higher proportions of high SES agents, which might be difficult to find, particularly if the city has marked residential segregation. In fact, with fewer agents of lower status around initially, higher SES agents are then more willing to visit UGS, which subsequently drives an overall increase in visits, but at the expense of inequality in visiting levels (Fig. 6 right-hand plots). This phenomenon has interesting implications for policy and practice, as it suggests unanticipated consequences of encouraging different SES groups to visit UGS. In particular, sharply increasing the willingness of lower SES agents to visit UGSs might produce a retreat on the part of those of higher SES, triggering a decline in overall visits. A reduction in overall visits was observed for higher levels of *ht*, in particular for the highest values of *t* (rightmost quadrants in left diagrams, Fig. 6). In this case, when fewer heterophilic low SES agents visit UGS, there is no corresponding increase in other groups, who are already visiting UGS with a frequency close to the theoretical maximum.

## 6 Plausibility of model output: a between-model comparison

In this study, we built an ABM to assess the plausibility of theory about what influences visits to UGS. We did not have detailed data about the decision-making processes that individuals undertook, nor did we have granular, spatially precise, longitudinal data on visiting behaviour itself. If such data were available, we would not have needed to create a simulation. Modelling in this way poses a key challenge; how can we be confident that the ABM is plausible and valid? Grimm (2005) introduced the concept of pattern-oriented modelling, where agent-based models of complex systems are developed and validated by the matching of multiple patterns simultaneously. The ability of the model to broadly reproduce observed patterns of UGS visitation, discussed in the paragraphs above, can be considered an initial form of pattern matching. In the absence of data against which to validate, one further comparison pattern can be generated by an alternative simulation approach, spatial microsimulation.

Spatial microsimulation involves “the creation and analysis of individual level data allocated to geographic zones” (Lovelace and Dumont, 2016). It is a relatively conventional technique, well-tested in the fields of health care demand (Clark et al., 2017; Campbell and Ballas, 2016), consumption patterns (James et al., 2019), and population projections among many others. The technique creates a synthetic dataset of spatially referenced individuals whose characteristics, when aggregated, match known values for their area of residence. The UK Census 2011 tells us the total number of people in small spatial areas by age, gender, ethnicity and SES. However, these are area-level totals only; the census does not provide the information at individual level. Further, the census does not ask questions about visits to UGS. SPANS provides data at an individual level, including age, gender, ethnicity and SES, and visits to UGS, but only for a sample population and with very little geographical specificity. Spatial microsimulation borrows information from each data source to generate the ideal; data at individual level, including the behaviour of interest, and at a fine geographical scale.

**Fig. 7:**
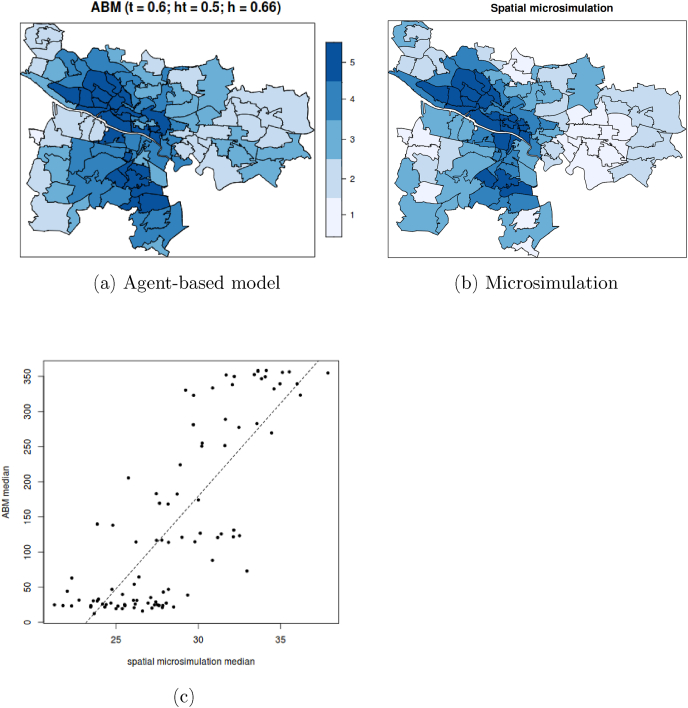
Distribution of UGS visitation in Glasgow by agents' area of residence. The cartograms show the quintile of each Detailed Postcode sector in the spatial microsimulation model, and in the ABM under a specific parameter combination (*t* = 0.6; *ht* = 0.5; *h* = 0.66). Plot (c) shows the relation between the median number of visits in the ABM and the MSM models. Each point in the scatterplot is a Postcode sector in Glasgow. Pearson's correlation is 0.88.

We undertook a spatial microsimulation of Glasgow, combining census data for datazones (small areal units) and the SPANS respondents who were resident in Glasgow. The simulated population was fitted and constrained by 4 variables: age, gender, ethnicity and SES. The sample of Glasgwegians surveyed in SPANS was representative of the city's population. In effect, this process created a dataset of individuals, ‘borrowed’ from SPANS, whose aggregate characteristics matched the real population in each small area. Since the individual dataset stems from SPANS, it also includes the ‘frequency of visits to UGS′ variable. The MSM output can be interpreted as the spatialisation of the regression model presented in the Supplementary material. The cartograms in Fig. 7 map the spatial distribution of frequency of visits to UGS emerging from the ABM (a) and compares it to that derived from the microsimulation (b). The areas of the city characterised by higher and lower UGS visitation are similar between the two models. In Fig. 7c, for each Postcode sector, we plot the relationship between the median number of visits produced in the ABM and in the MSM. While the ABM generates a significantly higher number of visits and a slightly more polarised distribution, the values produced in the two simulations are strongly correlated (Pearson's *r* = 0.86).

The relatively strong relation between the two model outputs signals that the patterns produced in the ABM are at least consistent with those suggested by the microsimulation. This was not a validation in the strictest sense, as both datasets are synthetic, but it provided reassurance that the agents in the ABM choose to behave in a way that, in aggregate, matches what the best available data describe.

## 7 Discussion

We created a stylised, spatially explicit, ABM to assess the plausibility that intra-city socio-economic inequalities in visiting UGS, and inter-city differences in visit levels and inequalities, could be produced from interactions between the socio-spatial structure of cities and attitudes of residents to the socio-economic mix of UGS visitors. Our hypothesis was that patterns of UGS visiting are the result of a complex interaction of urban environment, socio-spatial segregation, and - crucially - adaptive behaviour by individuals. In what was, essentially, a sophisticated thought experiment (Di Paolo et al., 2000), we designed a minimalist decision making mechanism implementing inter-group tolerance thresholds, and evaluated – given the real-world context of Scottish cities, their population and green space distributions – its ability to reproduce patterns observed in empirical data describing the issue of inequalities in UGS usage.

The inter- and intra-city socioeconomic inequalities in visiting UGS, and inter-city differences in visit levels observed in real world data, emerged jointly in the simulation under reasonable behavioural assumptions. This indicates that the interaction between the socio-spatial structure of cities and attitudes of residents to the socio-economic mix of UGS visitors can be a contributing factor to the emergence of inequalities. We were reassured that the model was plausible by both comparison with alternative simulations, and the model's consistent reproduction of empirically observed patterns. We then used the model to assess the sensitivity of the patterns to behavioural attitudes of the agents and to the influence of walkability and park quality.

The system we designed was primarily driven by interaction between agents of different SES, each of which adapts to the presence of others by choosing whether to use a certain park and, in doing so, contributes to shaping the social environment of other agents who, in turn, modify their behaviour again, adapting to the new situation. The implications of the socio-spatial arrangement of Scottish cities, in combination with behaviours, would be very hard to predict without the cognitive extension of a simulation (Bedau, 1997). Analysing the simulation output we were also able to tell that, under its behavioural rules, the main factors that determine the phenomena were primarily the proportion of high-status agents in a city and their spatial segregation from the rest of the population. The urban environment also plays against equity, with agents of higher status generally more likely to enjoy the benefits of walkability, and agents of lower status penalised by lack of maintenance of local green spaces.

### 7.1 Significance for public health

This study offers two key messages for those focused on the intersection between public health and the management / promotion of UGS. First, it supports the idea in the literature that issues related to social integration, both in the sense of spatial integration and perception and acceptance of others, are at the roots of the currently observed inequalities in green space usage. This, in turn, suggests that *simple* interventions aimed at encouraging certain groups to engage more with green space may have unanticipated consequences for population levels of, and inequalities in, UGS visits. The model suggests that they could even prove counterproductive, perhaps triggering withdrawal in other groups. Second, this study can serve to guide further investigation into the dynamics of engagement with nature: future quantitative surveys should include questions related to the perception and attitudes towards other green space users, to corroborate or disprove the suggestions of this work. For researchers working with more conventional statistical approaches, this study suggests that it is important to control for the socio-spatial structure of the study region. We observed that the interaction between neighbourhood segregation and the location of UGS was important in explaining use patterns. These factors are seldom attributed relevance in analyses explaining UGS visiting behaviour, but our model shows they may have a key role in shaping the dynamics.

### 7.2 Limitations

While the model was able to reproduce the salient characteristics of the phenomenon of interest, there were features of the observed reality which did not emerge accurately in the model. The absolute number of visits emerging in the model was distant from the observed one, so was the distribution of individual visits to UGS, and the proportions between cities are not reproduced accurately in the simulation. In particular, visits to UGSs in Dundee tend to be systematically underestimated when compared to the starting dataset. This was perhaps due to the relative scarcity of high SES agents there. Visits in Aberdeen, on the contrary, were overestimated, due to the greater number of high SES agents. These discrepancies are not unexpected. The stylised model presented here, assuming only walking trips to parks and a minimalist behavioural mechanism, was not designed to account for the whole range of people's motivations and constraints in visiting UGS, nor to reproduce observed patterns to the letter, or make point predictions. The purpose of this modelling exercise was to establish whether inter-group preferences could, in principle, produce socioeconomic and spatial differences in UGS visitation similar to those observed in reality. We demonstrated that these were, in fact, *sufficient* to reproduce the emergence of patterns qualitatively similar to those observed in the real world. Still, a sufficient explanation is not a necessary one, as a property of multi-level systems is equifinality, or multiple realisability. A macro-level is multi-realisable when it can be implemented in different ways. Inevitably, an ABM generates the higher-level effect by following one of the possible generating paths (Conte and Paolucci, 2014).

Despite the model's limitations, the findings presented here highlight certain mechanisms which is plausible to believe contribute to shaping the dynamics we observe in the real world. The ambition of stylised models of this sort is to provide insight into phenomena, offer a different, less explored angle, just as the forefather of this class of simulations did (Schelling, 1971b).

## 8 Conclusion

The broader socio-spatial arrangement of cities, and the preferences of individuals for sharing (or not) space with other similar types of people can combine, in a complex system, to create both inter- and intra-city inequalities in the use of UGS. Simple interventions based on providing more green space, or promoting its use, could plausibly exacerbate these inequalities. We urge more attention on the social and spatial context of UGS as we attempt to increase and equalise its use. Our study shows that ABMs are a useful way to integrate different kinds of knowledge; qualitative and quantitative, in the understanding of complex socio-spatial phenomena.

## Funding

This work was supported by the UK Medical Research Council (grant numbers MC_UU_00022/4 and MC_UU_00022/1) and the Scottish Chief Scientist Office (grant numbers SPHSU16 and SPHSU19).

## Footnotes

1 Full model source code and accompanying datasets can be downloaded from the following URL: https://github.com/harrykipper/MUGS.

2 It would be an equivalent formulation to state that heterophilic agents accept a maximum proportion *ht* of agents of their own SES. Therefore *ht* can either be thought of as the maximum accepted proportion of those in their own SES, or the minimum desired proportion of those in higher SES.

3 The model also displays intra-urban spatial inequality (not discussed here), with affluent areas of Glasgow registering very high rates of UGS visitation, comparable with those of Edinburgh, and less affluent areas with substantially less visitation.
