# Supplementary material for: Social integration as a determinant of inequalities in green space usage: Insights from a theoretical agent-based model
Source: Health Place. 2022 Jan;73:102729. doi: 10.1016/j.healthplace.2021.102729 (PMC8826000; doi:10.1016/j.healthplace.2021.102729)
Supplement: Multimedia component 1 [file mmc1.pdf]

Supplementary material

October 9, 2021

1 Scotland's people and nature survey 2014 - Exploratory regression analysis

Table 1: SPANS2014 - Only significant variables shown $adj.r^2 = 0.202$

	Estimate	Std.Error	t	Pr(>|t|)
age	-0.021687	0.003021	-7.180	1.09e-12 ***
ses C1	-0.399610	0.143350	-2.788	0.005376 **
ses C2	-0.673671	0.161398	-4.174	3.16e-05 ***
ses DE	-0.639118	0.169250	-3.776	0.000165 ***
has dog	2.127137	0.260502	8.166	6.67e-16 ***
has child	0.610248	0.126052	4.841	1.42e-06 ***
in Edinburgh	0.831809	0.151519	5.490	4.71e-08 ***
Owner	0.543732	0.136520	3.983	7.13e-05 ***
Renter	0.489021	0.171953	2.844	0.004516 **

Significance codes: 0 '***' 0.001 '**' 0.01 '*' 0.05 '.' 0.1 ' ' 1. Non significant variables include: gender, ethnicity, car ownership.

2 Population structure

Table 2: Proportion of SES

Local Authority	AB	C1	C2	DE
Aberdeen	0.24	0.34	0.22	0.21
Dundee	0.15	0.36	0.19	0.30
Edinburgh	0.30	0.37	0.14	0.20
Glasgow	0.17	0.32	0.18	0.33

3 Levels of segregation

Morrill index of segregation[1] of SES categories with Detailed Postcode sector spatial units. Edinburgh is the city with the lowest level of segregation of SES AB, Glasgow has the highest.

Table 3: Morrill index of segregation

Local Authority	AB	C1	C2	DE
Aberdeen	0.212	0.093	0.144	0.194
Dundee	0.216	0.067	0.090	0.147
Edinburgh	0.1295	0.069	0.181	0.178
Glasgow	0.255	0.062	0.134	0.159

4 Individual runs

Figure 1 shows the progression of median visits to UGS in each city throughout one simulation run. After initial turbulence, SES differenciation emerges during the first year, and stabilises before the end of the second year.

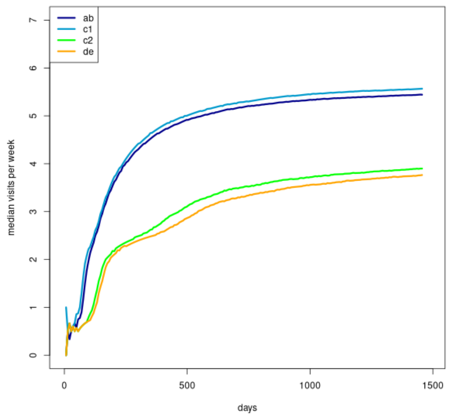

(a) Edinburgh

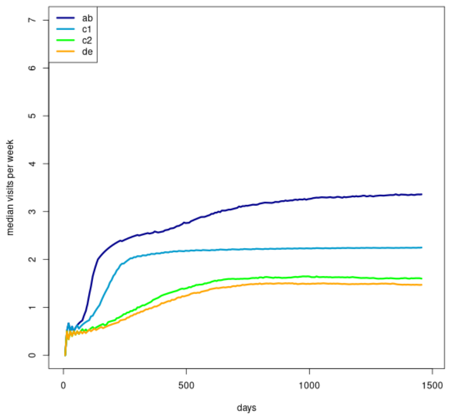

(b) Glasgow

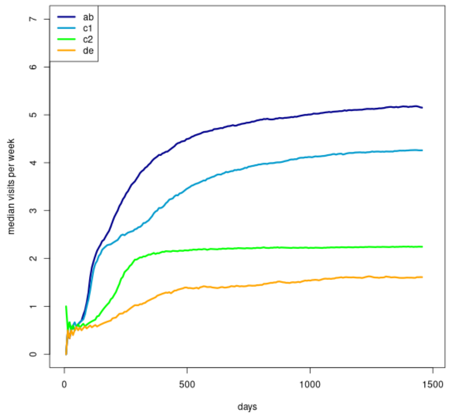

(c) Aberdeen

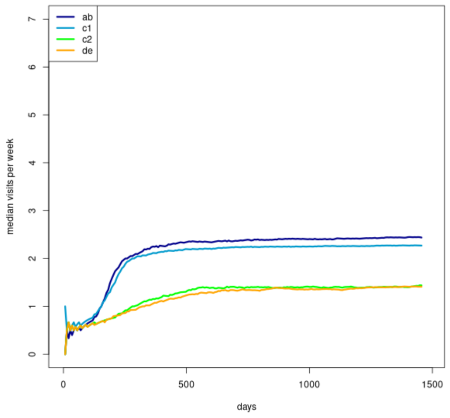

(d) Dundee

Figure 1: Median visits to UGS per SES and city across an individual run with $t = ht = 0.5$ and $h = 0.66$

5 Median visits to UGS in randomised runs

A set of experiments was performed in which the effect of socio-economic segregation is removed by randomising the location of agents within each city. Figure 2 shows median visits per SES in these runs, where we also assume $t = ht = 0.5$, i.e that all agents seek to visit UGS populated by a majority of agents of the desirable group. Without segregation, cities with a majority of high SES agents (Edinburgh and Aberdeen) accrue more visits, as high status agents (who are al-

ways homophilic) are nearly always surrounded by a majority of similar agents, especially for lower values of h, i.e when there are fewer heterophilic low status agents. In the only city with a majority of low SES agents, Glasgow, high SES agents visit substantially less times, as their homophilic preference is satisfied only occasionally. In this city low SES agents visit more, however their median number of visits stays low, as the fraction of heterophilic low SES agents is also frustrated by the scarcity of high SES agents. As this fraction increases, and more low SES agents seek the company of the scarce high SES agents, visits decrease for all agents.

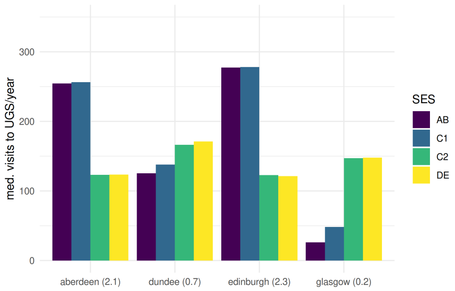

(a) $h = 0.33$

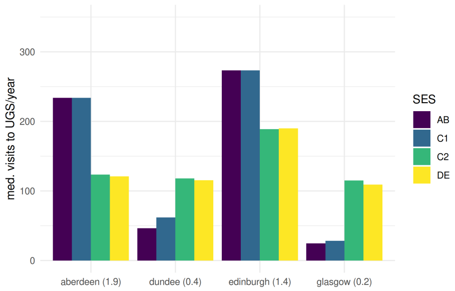

(b) $h = 0.5$

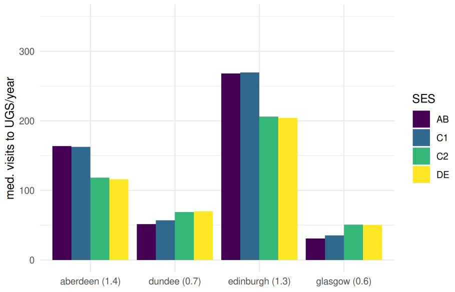

(c) $h = 0.66$

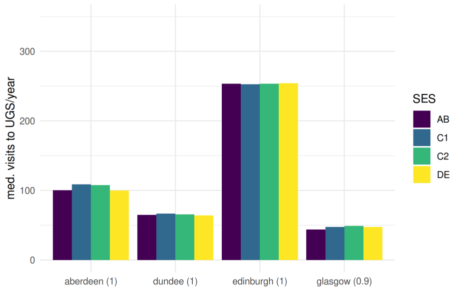

(d) $h = 1$

Figure 2: Median visits per SES per city at simulation end ($d = 1460$) for different values of h with $t = ht = 0.5$ and randomised population. Inequality shown in parentheses, expressed as the AB/DE proportion.

6 Impact of age differentials

Age difference as source of dissatisfaction is also implemented in the model. We assume that agents of 65 years of age or above are unsatisfied if the UGS is populated by 70% or more agents of age 30 or below. The impact of this constraint on the overall model behaviour is, however, limited. Figure 3 shows the parameter combinations giving rise to the observed phenomena when age is

considered as a dissatisfaction factor or not.

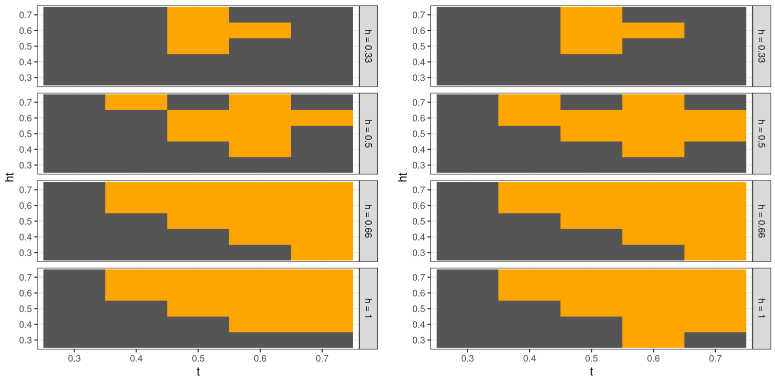

(a) no-age (b) age

Figure 3: In orange, parameter combinations giving rise to observed phenomena of Edinburgh excess and social gradients (conditions in Equations 1-3, Section 5, main paper) when age differentials are not considered (a) or considered (b).

7 Sensitivity analysis: impact of factor a

We performed sensitivity analysis on model parameter a. This value is a factor in the equations which adjust an agent's probability of visiting UGS after assessing other agents against the tolerance thresholds in a previous visit (Section 4.4, main paper). It represents the "intensity" of attitude change after a positive or negative experience at the UGS. In the main paper we set $a = 0.25$. Here we test values of $a = 0.01$, $a = 0.1$ and $a = 0.4$ with $t = ht = 0.5$, $h = 0.66$ including walkability and UGS quality. The diagrams below show the progression of visits across a model run under different values of a.

Among other things, factor a determines the speed by which the patterns in the model develop. Figure 4 shows that for the lowest tested value ($a = 0.01$) the dynamic of the model is only incipient in all four cities: the curves of median visits to UGS stay almost flat for all SESs for most of the simulation, starting to rise only towards the end. This is expected because when attitudes change at a very low pace the patterns emerge over a longer time frame.

For the tested values of $a > 0.01$, the dynamic of the model seems to be fairly constant, although it displays some sensitivity to the parameter. Specifically, we note that conditions 1-3 described in the main paper (Section 5) are verified slightly less frequently for $a \neq 0.25$ while confirming a trend for which these are found true more often for higher values of h and ht (Figure 5).

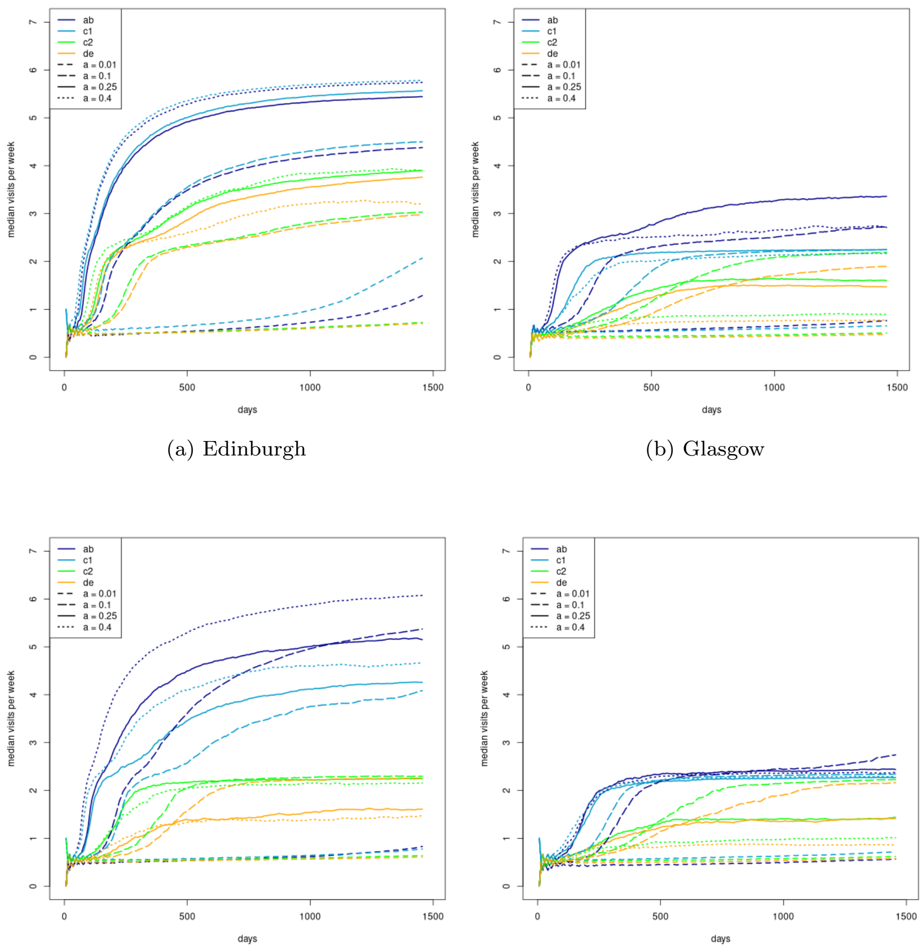

(a) Edinburgh (b) Glasgow

(c) Aberdeen (d) Dundee

Figure 4: Median visits to UGS per SES and city across an individual run for different values of a with $t = ht = 0.5$ and $h = 0.66$. Colour represents different SESs, line type differentiates between tested values.

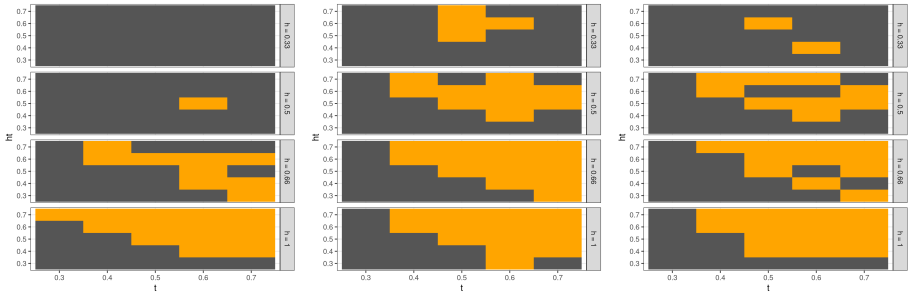

(a) $a = 0.1$ (b) $a = 0.25$ (c) $a = 0.4$

Figure 5: In orange, parameter combinations giving rise to observed phenomena of Edinburgh excess and social gradients (conditions in Section 6, main paper) for different values of a.

References

[1] R. Morrill. On the measure of geographic segregation. 2016.
