# Supplementary material for: Social integration as a determinant of inequalities in green space usage: Insights from a theoretical agent-based model
Source: Health Place. 2022 Jan;73:102729. doi: 10.1016/j.healthplace.2021.102729 (PMC8826000; doi:10.1016/j.healthplace.2021.102729)
Supplement: Multimedia component 2 [file mmc2.pdf]

Model description (ODD)

6th December 2021

1 Purpose of the model

Explore whether, and under what conditions, observed class and spatial differentials in urban green space visitation across Scottish cities can emerge from preferences on co-presence in green spaces based on class and age, together with environmental factors such as neighbourhood walkability and UGS maintenance levels.

2 Entities, state variables, and scales

Agents/Individuals

Agents represent individuals of 16 - 75 years of age. Agent attributes are listed in Table 3 below. Agents are explicitly represented and located, upon initialisation, on a random patch within their Postcode sector of residence, derived from Scotland's Census 2011. The location of residence is fixed and doesn't change throughout the simulation. Agents of Socio-economic status (SES) AB and C1 are considered "high status", C2 and DE are "low status".

Spatial units

A raster GIS representation of each Scottish city is superimposed on the simulated world. Each patch in the simulated world represents 625 square meters, although the scale is user-defined and can be changed in the model interface. The following location variables are imported from the GIS model:

- LSOA
- SIMD quintile
- DP sector
- isGreen

Locations with $isGreen = True$ belong to UGSs, all others are assumed to be residential and may house one or more agents. The place of residence of agents is fixed throughout the model.

Agents can access UGSs within their walking distance, but we assume that agents will be willing to walk more to reach larger UGS. In other words, UGSs have a 'catchment' proportional to their size (z). Agents are assigned the UGSs within reach in functions `set-preferred-parks` and `get-attractivity`, in the model source code according to the following table. Agents with no UGS within reach are assigned one, the closest, but their initial probability of visiting an UGS is reduced of a third.

Table 1: Catchment of UGS by size

size (z) in ha	distance walked
$z < 4$	200mt
$4 < z < 25$	500mt
$25 < z < 50$	750km
$50 < z < 225$	1km
$z > 225$	1.35km

Environment

The model implements certain environmental features which influence the mechanisms simulated. These are walkability, weather, UGS quality. Walkability is a patch attribute superimposed on the modelled world upon initialisation. It is derived from a GIS vector dataset obtained from the authors of MacDonald et el. [1].

UGS quality is determined upon initialisation and can be one of three levels: high, medium and low. We follow Ellaway and colleagues [2] who state that local green spaces in deprived areas tend to be less well maintained. We also assume that larger public parks and parks in city centres are always well maintained. The quality level is attributed to individual UGSs with the following steps, implemented in functions `create-parks` and `get-parkquality`, in the model source code:

1. Average size of the city's UGSs is determined
2. UGS of size above average are assigned quality High
3. UGS located in the city centre are assigned quality High
4. UGS of size below average located outside of city centre in areas of Deprivation quintile above the third are assigned quality High with probability 0.66 and quality Medium with probability 0.33
5. UGS of size below average located outside of city centre in areas of Deprivation quintile between 1 and 3 are assigned quality High with probability 0.33 and quality Medium with probability 0.66

6. UGS of size below average located outside of city centre in areas of Deprivation quintile 1 are assigned quality Medium with probability 0.33 and quality Low with probability 0.66

Agents react differently to UGS quality: we assume that all agents like UGS of quality High, and all agents have a probability 0.8 of disliking an UGS of quality Low. Agents of high SEP have a probability 0.65 of disliking an UGS of medium quality, while agents of Low SEP have a probability of 0.35 of disliking the same UGS (Table 2). These are implemented alongside threshold matching in function `iAmDissonant`, in the source code.

Table 2: Probability of dissatisfaction with UGS quality by SEP

UGS quality	AB,C1	C2,DE
High	0	0
Medium	0.65	0.35
Low	0.8	0.8

Time

One model tick represents one day, total run time is user-defined. Experiments in the paper last four years, or 1460 ticks.

3 Process overview and scheduling

A visual depiction of the step function is offered in Figure 1 below. At each time step agents are moved to a random patch belonging to the UGS at the top of their individual [accessible-parks] list, with probability $p_i \times w \times s$. Once all agents have made a decision and moved (or not), those who are in an UGS compute the SES proportion of a random 5% subset of other agents in the same UGS. The proportion is then compared with the agent's individual tolerance threshold (implemented in function `iAmDissonant`, in model source code). Attribute p is then recomputed according to satisfaction/non satisfaction. If the agent is unsatisfied, the park last visited is moved at the bottom of [accessible-parks]. The same happens if an agent over 65 years of age visits an UGS populated by 70% agents of age of 30 or below.

All agents then go back to their place of residence.

Every 7th step agents compare their frequency of UGS visits against that of a random agent x with age difference of 5 years or less $|age_x - age_i| \leq 5$ and SES difference ≤ 1 (e.g. agents of SES C1 will compare with one agent of similar age of SES AB, C1, or C2) residing in a 500 meter radius. If agent x has visited UGS 50% more or 50% less, agent i's propensity is adjusted in the direction of the neighbour as follows:

$$\begin{cases} p_i = p_i + (a \times p_i) & if\ v_x > (v_i \times 1.5) \\ p_i = p_i - (a \times p_i) & if\ v_x < (v_i \times 0.5) \end{cases}$$

Smaller discrepancies have no effect. This is implemented in function `getLocalInfluence` in the model source code.

4 Design concepts

Basic principles

The model follows the threshold/spatial cognitive dissonance approach. Agents are assumed to hold a preference for the company of agents of a certain group and adjust their behaviour according to the satisfaction of the preference after each interaction. Social conformity is also implemented in the model: agents tend to conform with their neighbours when large differences in UGS visitation exist (see Section 'Interaction', below).

Adaptation

Agents change their propensity to visit green spaces, and change the green space they visit (if more than one is accessible), according to the social and age composition of other visitors: whether the tolerance threshold towards members of the undesired group is / is not exceeded. They also adapt their willingness to visit UGSs to conform with neighbours of similar age and SES.

Sensing

Agents are assumed to be able to correctly detect the SES and age class of other agents when in an UGS.

Interaction

Interaction between agents is twofold.

1. When visiting an UGS agents react to the presence of others by checking the proportion of agents belonging to the two groups (high and low status) against their preference.

2. Agents are influenced by the habits of other agents of similar SES and age, residing in the same area. They select one random agent of similar SEP and age residing within 500 meters and compare their frequency of visits. In case of a wide discrepancy, the agent adjusts its probability of visiting an UGS conforming with the other agent as described above, in Section 3.

Stochasticity

The following attributes are assigned stochastically in each model run:

- exact location of agents within the Detailed Postcode area of residence.

- exact age of agents within the five-year interval derived from Census
- dog ownership. Proportion of dog owners by SES is based on Marsa-Sambola et al. [3]. The agents assumed to own dogs within each SES are determined stochastically upon initialisation.
- neighbouring agent to compare with in function `getLocalInfluence`. Determined stochastically every 7th step

Collectives

Agents belong to high (AB, C1) or low (C2, DE) SES. Their tolerance thresholds are based on the SES, the assumption being that agents are homophilic with various levels of tolerance for the other group, but a proportion of agents of low SES are heterophilic with a minimum desired amount of agents of the other SES.

5 Initialisation

- GIS files are read and the world is generated.
- Agents are read into the model from Scotland's Census 2011 datasets in function `read-agents`. The input files contain population counts by sex by age by socio-economic position, for each Detailed Characteristic Postcode Sector.
- Upon creation agents are assigned an initial probability of visiting an UGS in the subsequent time step: p. This is drawn from a normal distribution with $\mu = 0.07$ and $\sigma = 0.025$. Probability is then adjusted for age, assuming that agents above age 60 will visit less, using the following formula: $p_i = \frac{min((160-age),100)}{100} \times p_i$
- The catchment area of each UGS is determined per Table 1, and agents are assigned the UGS within reach, i.e. the list g is populated. The list will be empty for agents residing more than 1.35Km from the closest UGS. The list g of these agents is then populated with one UGS, the closest, and their probability is reduced by a third $p_i = p_i \times 0.66$

6 Input data

UGS are derived from OS Open Green space datasets; population data are derived from Scotland's census 2011. All datasets employed, along with model source code and the code used to perform the analysis and produce diagrams, are available at `http://github.com/harrykipper/MUGS`.

[1] https://www.currentresults.com/Weather/United-Kingdom/annual-sunshine.php

Table 3: Agent attributes

variable	description	type	range	source	held by
c	socioeconomic status	string	AB, C1, C2, DE	2011 Census	all agents
y	age	int	16 - 75	2011 Census	all agents
g	UGS accessible	list	$\{\dots\}$	GIS	all agents
p	prob. visiting UGS	real	0-1	hypothesis	all agents
t	homophily threshold	real	0-1	hypothesis	agents with $HA = 0$
ht	heterophily threshold	real	0-1	hypothesis	agents with $HA = 1$
HA	heterophilic agent	bool	0,1	hypothesis	all agents
v	# visits to UGSs	int	0 - 1460	model output	all agents
d	dog owner?	bool	0,1	[3]	all agents

Table 4: Environment variables

variable	description	type	range	source
s	weather	real	0 - 1	Current results website [1]
h	proportion of agents of C2 and DE SEP with $HA = 1$	real	0 - 1	hypothesis

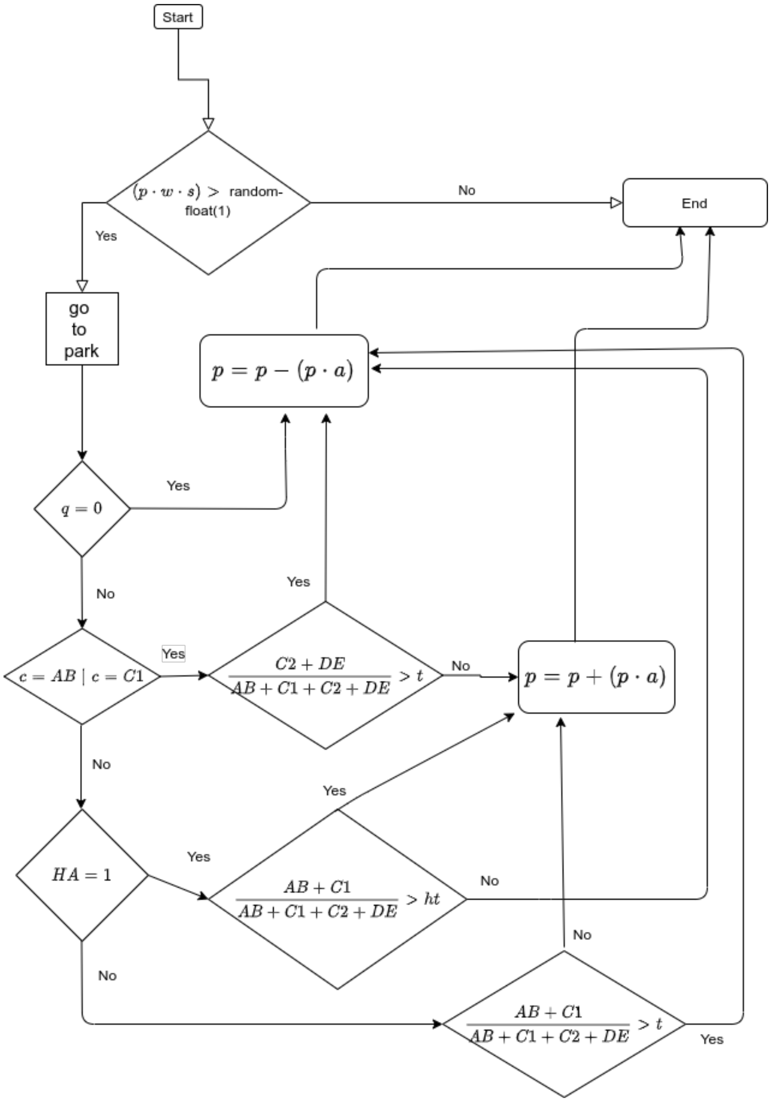

Figure 1: Simulation step

References

[1] L. Macdonald, P. McCrorie, N. Nicholls, A. Ellaway, Walkability around primary schools and area deprivation across Scotland, BMC Public Health 16 (1) (2016) 1–7. doi:10.1186/s12889-016-2994-0.
URL http://dx.doi.org/10.1186/s12889-016-2994-0

[2] A. Ellaway, A. Kirk, S. Macintyre, N. Mutrie, Nowhere to play? The relationship between the location of outdoor play areas and deprivation in Glasgow, Health & Place 13 (2) (2007) 557–561. doi:10.1016/j.healthplace.2006.03.005.
URL https://linkinghub.elsevier.com/retrieve/pii/S1353829206000256

[3] F. Marsa-Sambola, J. Williams, J. Muldoon, A. Lawrence, M. Connor, C. Roberts, F. Brooks, C. Currie, Sociodemographics of Pet Ownership among Adolescents in Great Britain: Findings from the HBSC Study in England, Scotland, and Wales, Anthrozoos 29 (4) (2016) 559–580. doi:10.1080/08927936.2016.1228756.
